# Antibiotic Use: A Cross-Sectional Survey Assessing the Knowledge, Attitudes and Practices amongst Students of a School of Medicine in Italy

**DOI:** 10.1371/journal.pone.0122476

**Published:** 2015-04-01

**Authors:** Giacomo Scaioli, Maria R. Gualano, Renata Gili, Simona Masucci, Fabrizio Bert, Roberta Siliquini

**Affiliations:** Department of Public Health, University of Turin, Turin, Italy; University of Chieti, ITALY

## Abstract

**Background:**

Since antibiotic resistance has become a worldwide public health concern and is in part related to physicians’ lack of knowledge, it is essential to focus our attention on healthcare profession students. The present study aims at evaluating the knowledge and attitudes of the School of Medicine’s students towards antibiotic usage and antibiotic resistance.

**Methods:**

In December 2013, a cross sectional study was conducted amongst medical, dental, nursing and other health care profession students of the School of Medicine at the University of Torino. Students of all the academic years took part in this study. Questionnaires were submitted during regular lectures (only students who attended courses on one specific day were surveyed) and the data collected was analyzed using StataMP11 statistical software.

**Results:**

Overall, 1,050 students were interviewed. The response rate was 100%. Around 20% of the sample stated that antibiotics are appropriate for viral infections and 15% of the students that they stop taking those drugs when symptoms decrease. Results of the multivariate analyses showed that females were more likely than males to take antibiotics only when prescribed (OR 1.43, 95% CI 1.04–1.98). Interestingly, students with a relative working in a health-related field, as well as those who took at least one course of antibiotics in the last year, had a lower probability of taking those drugs only under prescription (OR = 0.69 95% CI: 0.49–0.97 and OR = 0.38 95% CI: 0.27–0.53, respectively).

**Conclusion:**

The present paper shows how healthcare profession students do not practice what they know. Since those students will be a behavioral model for citizens and patients, it is important to generate more awareness around this issue throughout their studies. It would be advisable to introduce a specific course and training on antibiotics in the core curriculum of the School of Medicine.

## Introduction

The threat of antibiotic resistance has become a worldwide public health concern, with a substantial economic and clinical burden. The World Health Organization (WHO) estimated that this problem leads to an excess of mortality of 25,000 people every year in the European hospitals, with a cost of about 1,5 billion of Euro [1].

Antibiotics are the most frequently prescribed drugs, but they are often misused [2,3]. This contributes to the spreading of resistant strains of bacteria [4]. One of the causes for the antimicrobial misuse is linked to a wrong prescribing behavior amongst physicians [5–7]. There are many factors which could influence doctors’ decisions, leading them to breach the principles of a good clinical practice. For example, the fear of possible future complications in their patients, or a desire to fulfill patients’ expectations [1,8]. Patients’ wrong habits and their lack of knowledge may also represent another leading cause for antimicrobial resistance [1,9–14].

Educational initiatives on the correct use and prescription of antimicrobial drugs, addressed to both the general population and health care professionals, should thus be promoted [15]. In particular, it would be desirable to focus on the new generations of health care professionals[16–17]. Indeed, they must be fully aware of the increasing problem of antibiotic resistance, since they will be the future antibiotic providers [1,16,17].

Several studies have tried to measure the knowledge, attitude and behavior of medical students towards antibiotics. The majority of these studies have a relatively low sample size, and in three of them the response rate was lower than 50%. All these studies were performed in a single country (China, Democratic Republic of Congo, France, India, UK, USA and Jordan), while one study was carried out in seven European countries. Moreover, the existing literature is mainly focused on antibiotic prescribing behaviors rather than on attitudes about antibiotic consumption. Although the results of these studies are in some cases encouraging, in many others they show a lack of knowledge on the importance of a correct antibiotic use and prescription [18–26].

Up until now, in Italy, the only study assessing the knowledge and attitudes towards antimicrobial drugs was conducted on a sample representative of the general population, while works concerning healthcare profession students are lacking [27]. Italy is a Country with a high consumption of antibiotics. Data from the “Antimicrobial consumption interactive database” of the European Center for Disease Prevention and Control (ECDC) showed how, in 2011, Italy was in sixth place in Europe in terms of antibiotic consumption pro capita [28]. Moreover, the percentage of most common bacteria resistant to at least one antibiotic is higher than the European average, according to the European Antimicrobial resistance network [29]. In this contest, Piedmont (the region whose capital is Torino), registered one of the lowest consumption of antimicrobial drugs in Italy [30]. Nevertheless, this consumption is still high if compared with other European countries [28].

In this scenario, the objective of the present study is to evaluate the knowledge and attitudes of healthcare profession students about antibiotic usage and antibiotic resistance for the first time in Italy.

## Materials and Methods

In December 2013, a cross-sectional survey was carried out amongst a convenience sample of medical, dental, nursing and other health care profession students (dental hygienists, orthoptists, speech therapists, childhood neuro and psycomotricity therapists, physiotherapists) attending the School of Medicine of Torino, one of the largest Italian Universities. Two trained doctors of the School of Public Health at the University of Torino administered the questionnaires to students attending regular class sections during one specific day. The day of the submission of the questionnaire varied on the basis of the degree course and the year of study. Students interviewed represented around 30% of the total students of the School of Medicine of Torino.

Participation was voluntary, anonymous and without compensation. The researchers assured that anonymity would be maintained, and ethical principles would be followed. Before the administration of questionnaires, the background and intentions of the survey were explained, and students were encouraged to participate without any undue pressure. The study was approved by the Institutional Review Board of the Department of Public Health Sciences of University of Torino. Returning of the completed survey was accepted as consent by the participating students.

### The questionnaire

A 31-point self-administered questionnaire, composed by five sections, was used (S1 Questionnaire). The questionnaire was developed after a literature review of comparable studies and previously validated by a pilot study on 20 medical students [18– 25,31–34].

The first part of the questionnaire investigated socio-demographic characteristics of the students interviewed, such as age, gender, place and year of birth and type of degree.

The second part was addressed to evaluate the consumption of antibiotics in the last year.

In the third part the knowledge about antibiotic and related adverse reaction was assessed, while in the fourth part information about awareness of antibiotic resistance was collected. Finally, the last part focused on attitudes and behaviors towards antibiotic use. Both a 4-point Likert-scale, whose responses ranged from “Strongly disagree” to “Strongly agree”, and dichotomous answers (yes/no) were used [35].

### Statistical analysis

The results were analyzed using the StataMP11 statistical software (Stata Corp., College Station, TX, 2011). Firstly, a descriptive analysis of the sample was conducted, considering the distribution of gender, age, country of birth, relatives working in health-related field, and degree course. Results were expressed in frequencies and percentages for dichotomous variables and mean with standard deviation (SD) for continuous variables.

The outcomes concerning knowledge were firstly described with absolute numbers and percentages, then dichotomized as “correct” versus “incorrect”, grouping the 4-point Likert scale as follows: “totally agree” and “agree” (as correct), versus “totally disagree” and “disagree” (as incorrect). The appropriate answer could be “Strongly agree/Agree” or “Strongly disagree/Disagree” depending on the meaning of the question. For each outcome, the percentages of appropriate answers were calculated.

Six outcome variables about attitudes were considered:

- Do you take antibiotics only when prescribed by the doctor?
- Do you have leftover antibiotics at home?
- Do you usually buy antibiotics without medical prescription?
- Do you usually take antibiotics after a simple phone call with your doctor without a proper medical examination?
- Do you usually stop taking antibiotics when you start feeling better?
- Do you usually use leftovers antibiotics without consulting a doctor?

For each of these outcomes logistic regression models were then carried out in order to assess the role of socio-demographic variables. The covariates to be included into the final model were selected using a stepwise forward selection process, with a univariate p-value <0.25 as the main criterion [36], with age and gender as potential confounders. Results are expressed as Odds Ratio (OR) with 95% Confidence Intervals (CI), and a two-tailed p-value ≤0.05 was considered significant for all analyses.

## Results

### Characteristics of the sample

A total of 1,050 students were asked to participate and the response rate was 100%. Students of all the academic years took part in this study. The overwhelming majority of participants (96.7%) were born in Italy. Among foreign students, around 50% were from eastern European Countries (Albania, Moldavia, Romania, Belarus) and around 20% from Africa (Cameron, Morocco, Burkina Faso). Females were 59.6% of the sample. The mean age was 20.98 (SD ±2.94). Almost one-third of the participants (30.6%) declared to have at least one relative that works in a health-related field (Table 1).

**Table 1:** Description of the sample.

		N (%)
**Gender**	Female	620 (59.56)
	Male	421 (40.44)
**Age**	-	20.98 (±2.94)^a^
**Country of birth**	Italy	1012 (96.66)
	Foreign countries	35 (3.34)
**Relatives working in health related field**	Yes	320 (30.65)
	No	724 (69.35)
**Degree course**	Medicine	768 (73.28)
	Health professions	241 (23.00)
	Dentistry	39 (3.72)
**Year of study**	First	465 (44.28)
	Second	187 (17.81)
	Third	190 (18.09)
	Fourth	70 (6.67)
	Fifth	79 (7.52)
	Sixth	59 (5.62)
**Antibiotic use in the last year**	Yes	478 (45.6)
	No	570 (54.4)

(N = 1,050).

^a^ mean (Standard Deviation)

Less than half of the sample (45.6%) claimed to have taken antibiotics in the last year. Of these, 81.8% stated to have taken only one course of these drugs, 15.5% two courses and 2.7% three or more courses.

### Knowledge about antibiotics

The participants demonstrated a fair good knowledge about antibiotics. Percentages of fully correct answers (“strongly agree” or “strongly disagree” depending on the statement) were higher than 80% in one third of the statements proposed. The lowest percentage was reached for the statement “Antibiotics can cause secondary infections after killing good bacteria present in our organism” and only 40% of the students strongly agreed (S1 Table).

After dichotomizing the 4-point Likert scale, it was noticed that more than 90% of the sample answered correctly to the statements “Penicillin and amoxicillin are antibiotics” (96%), “Aspirin is an antibiotic” (98.9%), “Paracetamol is an antibiotic” (96.4%).

Regarding the knowledge about antibiotic use, almost all the participants were aware that antibiotics are useful for treating bacterial infections (95.2%) and that these drugs are not indicated for every kind of pain and inflammation (96.6%). Moreover, a relatively low percentage of the sample (83.2%) was conscious that antimicrobial drugs are not appropriate for viral infections.

In the matter of knowledge of antibiotics’ side effects, more than 90% of the participants agreed that “Antibiotics can kill “good bacteria” present in our organism” and “Antibiotics can cause allergic reactions” (90.2% and 93.4% respectively), while four-fifth of the sample (79.5%) stated that antimicrobial drugs can cause secondary infections.

The three last statements were related to antibiotic resistance knowledge. The overwhelming majority of participants were aware that “Antibiotic resistance is a phenomenon for which a bacterium loses its sensitivity to an antibiotic” (93.9%) and that “Misuse of antibiotics can lead to antibiotic resistance” (98%). Moreover, 94.8% of the students interviewed knew that it is mandatory to finish the full course of antibiotics even if the symptoms are improving.

### Attitudes and behaviors about antibiotics

Concerning attitudes and behaviors on antibiotic consumption, almost all the students interviewed stated they usually do not take antibiotics for flu or cold or a sore throat. Interestingly, 15.2% of them declared to stop taking these drugs when symptoms decrease, and 17.7% that they normally use leftovers antibiotics without consulting a doctor. Moreover, 16% of the sample claimed to buy antibiotics without medical prescription, and 36.6% usually start taking antibiotics after a simple phone call with the doctor, without a proper medical examination (Table 2).

**Table 2:** Attitudes and behaviors about antibiotic consumption (N = 1,050).

		N(%)
Do you usually take antibiotics for cold or sore throat?	Yes	4 (0.38)
	No	1044 (99.62)
Do you usually take antibiotics for fever?	Yes	19 (1.81)
	No	1029 (98.19)
Do you usually stop taking antibiotic when you start feeling better?	Yes	158 (15.22)
	No	880 (84.78)
Do you take antibiotics only when prescribed by the doctor?	Yes	852 (81.38)
	No	195 (18.62)
Do you have leftover antibiotics at home?	Yes	648 (62.01)
	No	397 (37.99)
Do you use leftovers antibiotics when you have cold, sore throat or flu without consulting your doctor?	Yes	185 (17.70)
	No	860 (82.30)
Do you buy antibiotics without medical prescription?	Yes	167 (16.04)
	No	874 (83.96)
Do you start taking antibiotics only after a phone call with your doctor?	Yes	375 (36.06)
	No	665 (63.94)

Since two questions analyzed the students’ inclination to buy and take antibiotics without medical prescription, results of the logistic regression models showed how females had a significant higher likelihood of taking antibiotics under prescription, if compared to males (OR = 1.43, 95% CI: 1.04–1.98). Instead, students with a relative working in a health-related field and those who took at least one course of antibiotics in the last year seem to consider medical advices not strictly necessary to take antimicrobial drugs (OR = 0.69, 95% CI: 0.49–0.97 and OR = 0.38, 95% CI: 0.27–0.53, respectively). Lastly, the longer the time spent at Medical School, the more were students prone to buy antibiotics without medical prescription (OR = 1.18, 95% CI: 1.04–1.34) and the less were students likely to take only prescribed antibiotics (OR = 0.84, 95% CI: 0.75–0.95).

Two other statements examined students’ propensity to store leftovers antibiotics at home and to use them without consulting a doctor. In this regard, participants with a relative working in a health-related field had a higher probability to keep antimicrobial drugs in their houses (OR = 1.80, 95% CI: 1.34–2.42), while those who took antibiotics in the last year are more likely to consume leftovers antibiotics without physician’s advices (OR = 1.75, 95% CI: 1.27–2.42) (Tables 3 and 4). Interestingly, a logistic regression including the degree course as a variable (S2 Table), showed how health professions students had a lower probability than medical students to keep leftover antibiotics at home and to take antibiotics after a simple doctor call, without a proper medical examination (OR = 0.64, 95% CI: 0.47–0.87 and OR = 0.60, 95% CI: 0.43–0.83, respectively).

**Table 3:** Multivariate results on attitudes and behaviors about antibiotic consumption (N = 1,050).

		Do you take antibiotics only when prescribed by the doctor?		Do you have leftover antibiotics at home?		Do you usually buy antibiotics without medical prescription?	
		OR (95% CI)	p	OR (95% CI)	p	OR (95% CI)	p
Gender	Male	1		1		1	
	Female	1.43 (1.04–1.98)	0.03	0.90 (0.69–1.18)	0.65	0.94 (0.66–1.33)	0.73
Age^a^		1.01 (0.95–1.07)	0.76	0.92 (0.87–0.96)	<0.01	0.97 (0.90–1.04)	0.46
Country of birth	Italy	-		1		-	
	Foreign	-		0.83 (0.41–1.69)	0.66	-	
Relatives working in health-related field	No	1		1		-	
	Yes	0.69 (0.49–0.97)	0.03	1.80 (1.34–2.42)	<0.01	-	
Year of study^a^		0.84 (0.75–0.95)	<0.01	-		1.18 (1.04–1.34)	0.01
Use of antibiotic in the last year	No	1		1		1	
	Yes	0.38 (0.27–0.53)	<0.01	2.40 (1.84–3.13)	<0.01	2.87 (2.02–4.08)	<0.01
Have you ever heard about antibiotic resistance	No	-		1		-	
	Yes	-		0.71 (0.39–1.30)	0.21	-	

^a^ “Age” and “year of study” are considered as continuous variables.

**Table 4:** Multivariate results on attitudes and behaviors about antibiotic consumption (N = 1,050).

		Do you usually take antibiotics after a simple phone call with your doctor without a proper medical examination?		Do you usually stop taking antibiotics when you start feeling better?		Do you usually use leftovers antibiotics without consulting a doctor?	
		OR (95% CI)	p	OR (95% CI)	p	OR (95% CI)	p
Gender	Male	1		1		1	
	Female	1.05 (0.81–1.37)	0.42	0.93 (0.66–1.32)	0.70	0.95 (0.68–1.32)	0.75
Age^a^		0.99 (0.94–1.04)	0.99	0.96 (0.89–1.04)	0.32	0.98 (0.91–1.05)	0.57
Country of birth	Italy	1		1		-	
	Foreign	0.58 (0.25–1.32)	0.19	2.47 (1.09–5.61)	0.30	-	
Relatives working in health-related field	No	1		-		-	
	Yes	0.67 (0.50–0.89)	<0.01	-		-	
Year of study^a^		-		0.94 (0.81–1.09)	0.44	1.11 (0.98–1.25)	0.09
Use of antibiotic in the last year	No	1		1		1	
	Yes	1.85 (1.42–2.40)	<0.01	1.82 (1.28–2.57)	<0.01	1.75 (1.27–2.42)	<0.01
Have you ever heard about antibiotic resistance	No	1		1		1	
	Yes	0.53 (0.31–0.91)	0.02	0.64 (0.33–1.23)	0.18	0.58 (0.30–1.09)	0.09

^a^ “Age” and “year of study” are considered as continuous variables.

Moreover, the last two statements highlighted how having a relative working as health professional and being aware of the problem of antibiotic resistance seem to decrease the likelihood of taking antimicrobial drugs without a proper medical examination (OR = 0.67, 95% CI: 0.50–0.89 and OR = 0.53, 95% CI: 0.31–0.91 respectively) and how foreign students had a higher risk of interrupting the course of antibiotics as soon as symptoms improve (OR = 2.47, 95% CI: 1.09–5.61) (Table 4).

## Discussion

This study aims at assessing the knowledge and attitudes of Italian healthcare profession students towards antibiotics. To our knowledge, in Italy, only one published study investigated those topics amongst the general population but no one focused on healthcare students [27].

Our findings showed how the students interviewed had a fair good knowledge about the role of antibiotics, their consumption and the related adverse reactions. More than 90% of the sample answered correctly to all the items administered, with two important exceptions. Indeed, around one fifth of the sample was not aware that antimicrobial drugs are not effective against viruses and that antimicrobial drugs can cause secondary infections.

These results are confirmed by recent studies on this topic. A survey performed in US showed how almost all the medical students interviewed were aware that inappropriate use of antimicrobials can harm patients and cause antibiotic resistance [25]. A European multicentre study pointed out how the 80% of medical students believed that antibiotic resistance was a problem in their own hospital [21]. Moreover, a survey performed in the Democratic Republic of Congo demonstrated that 87% of the medical students and medical doctors think that antibiotics are overused in that Country [19].

Furthermore, the results of the present study are encouraging if compared to the Italian general population. The 2013 Eurobarometer report on antibiotic resistance pointed out how the 58% of the Italian subjects interviewed believed that antibiotics can kill viruses, while the 40% of the sample stated that these drugs are effective against cold and flu [13]. These results are partially confirmed by a study performed on Italian general population by Napolitano et al. In this case, around half of the sample was not aware that antibiotics are not useful for flu, fever, and sore throat [27]. On an international level, a meta-analysis of 24 studies on this topic reported comparable results [14].

Despite the fair good level of knowledge, high rates of incorrect behaviors were noticed. Hence, it seems like despite having an sufficient theoretical background, School of Medicine’s students do not practice what they learn. Indeed, more than 15% of the sample declared to stop taking antibiotics when symptoms improve and to use leftover antibiotics without consulting a doctor.

Two other studies that assessed attitudes in antibiotic use among health profession and medical students, Khan et al. and Suaifan et al., showed even less promising results than those presented in the present paper, probably due to the different socio-cultural context [22,24]. Indeed, the Indian paper pointed out how about 20% of the students interviewed always interrupt the antibiotic course if they start feeling better and give leftovers antibiotics to their friends or roommates [22]. In a study carried out in Jordan, more than 60% of the respondents declared they did not complete the last course of antibiotics [24].

In the general population, a recent Italian survey showed how around 33% of the responders stated that they had taken an antibiotic without the prescription of a physician [27]. Moreover, a global survey performed by Kardas et al. showed how the 50% of the sample interviewed admitted having leftover antibiotics [37].

Logistic regression models showed interesting results in terms of identifying the most relevant socio-demographic factors influencing attitudes and behaviors of interviewed students. Students with a relative working in a health related field had a lower likelihood of taking antibiotics only when prescribed by the doctor and a higher probability to keep leftovers antibiotics at home. This is understandable, given that physicians often use to prescribe antibiotics at home as informal care addressed to their relatives and friends [38,39].

Moreover, the advancement within the degree curriculum could influence the choice of taking antibiotics without any medical consultation. Indeed, for every year spent at university there is a significantly lower probability of using antimicrobial drugs only when prescribed by the doctor, and a significant higher likelihood of buying these drugs without medical prescription. Students seem to be more confident with their knowledge once they attend the last years of university, with a higher risk of incoming in incorrect behavior [40]. Lastly, the present study demonstrated how females had a higher probability than males to use antibiotics only when prescribed by the doctor, showing an increased predisposition to avoid self medication with antibiotics. These results are partially in contrast with those pointed out in a recent study performed by Manteuffel et al., which demonstrated that women were less prone than men to receive chronic medications (such as drugs for diabetes and cardiovascular conditions), and to respect doctors’ recommendation when using medications [41].

This study presents several strengths. Firstly, it is the first study investigating this issue amongst healthcare professional students in Italy. Secondly, the size of the sample was higher than other studies on similar topic [20,22,25]. No students refused to complete the questionnaire, resulting in a high rate of responses.

However, the present paper had some limits that should be acknowledged, mainly related to the study design. For instance, a self-administered questionnaire was used, instead of the face-to-face interviews that are traditionally considered the gold standard method of survey administration [42,43]. Nevertheless, the administration of questionnaires during regular class sessions made it possible to have a higher response rate than the one usually obtained with telephone and e-mail surveys. Furthermore, the survey was conducted in a single university centre, in Torino, rather than opening up to different contexts, which could have given different results. Health care profession students of the University of Torino do not have a course specifically focused only on antibiotic resistance. This topic is discussed in several degree courses, such as a specific “Pharmacology” course in which antibiotics are deeply examined. Besides, the questionnaires were distributed during the regular class sessions, leading to a potential selection bias: students who attended university courses could be more motivated than ones who did not attend regular lectures. Moreover, in the first years, the percentage of courses attendance is higher than in the last years as students in the last years often prefer to attend hospital wards instead of attending class lessons.

Lastly, self-administered surveys could lead to a recall bias, caused by differences in the accuracy or completeness of the recollections retrieved by study participants, and to an under or over-reporting of respectively incorrect or correct behaviors and attitudes [44,45].

## Conclusions

The present paper demonstrated how medical and healthcare profession students do not practice what they know. The level of knowledge about antibiotics amongst the healthcare profession students is quite high but there are attitudes and practices that are still incorrect depending above all on having a relative working in a health-related field and on the increasing of the years spent at Medical School. Since the healthcare profession students will be a behavioral model for citizens and patients and once they will be medical doctors they will be able to prescribe these drugs, it is important to create more awareness on this topic during the degree courses. It would be advisable to introduce specific courses and training about antibiotics in the core curriculum of the Schools of Medicine, with more emphasis on the rules of right prescribing [1,16,17].

## Supporting Information

S1 Questionnaire 31-point self-administered questionnaire. (DOC)

S1 Table Knowledge about antibiotics. (DOC)

S2 Table Multivariate results on attitudes and behaviors about antibiotic consumption (including the variable “Degree course”). (DOCX)
