# Supplementary material for: Antibiotic Use: A Cross-Sectional Survey Assessing the Knowledge, Attitudes and Practices amongst Students of a School of Medicine in Italy
Source: PLoS One. 2015 Apr 1;10(4):e0122476. doi: 10.1371/journal.pone.0122476 (PMC4382153; doi:10.1371/journal.pone.0122476)
Supplement: S1 Questionnaire — (DOC) [file pone.0122476.s001.doc]

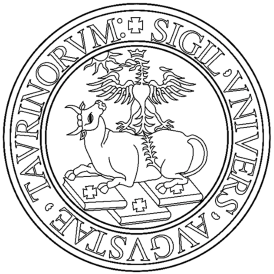


University of Torino

Department of Public Health Sciences and Pediatrics

**School**___________________________________ **Year of study** ___________

**First part: demographic data**

1 – Year of birth 2 – Gender: M F

3 –Place of birth (Country)

4 –Place of birth of the father (Country)

5 - Place of birth of the mother (Country)

6–At least one member of your family (parents, children, husband/wife) works in a health related field?

- yes
- no

**Second part: frequency of antibiotic use**

7–Have you used antibiotics in the last year?

- yes
- no

8–If yes, how many times?

- 1-2
- 3-5
- > 5

**Third part: knowledge about antibiotics**

9 - Penicillin or Amoxicillin are antibiotics.

| 1 | 2 | 3 | 4 |
| --- | --- | --- | --- |
| Total disagreement |  |  | Total agreement |

10–Aspirin is an antibiotic.

| 1 | 2 | 3 | 4 |
| --- | --- | --- | --- |
| Total disagreement |  |  | Total agreement |

11–Paracetamol is an antibiotic.

| 1 | 2 | 3 | 4 |
| --- | --- | --- | --- |
| Total disagreement |  |  | Total agreement |

12–Antibiotics are useful for bacterial infections (e.g. Tuberculosis).

| 1 | 2 | 3 | 4 |
| --- | --- | --- | --- |
| Total disagreement |  |  | Total agreement |

13 – Antibiotics are useful for viral infections (e.g. flu).

| 1 | 2 | 3 | 4 |
| --- | --- | --- | --- |
| Total disagreement |  |  | Total agreement |

14 - Antibiotics are indicated to reduce any kind of pain and inflammation.

| 1 | 2 | 3 | 4 |
| --- | --- | --- | --- |
| Total disagreement |  |  | Total agreement |

15 - Antibiotics can kill “good bacteria” present in our organism.

| 1 | 2 | 3 | 4 |
| --- | --- | --- | --- |
| Total disagreement |  |  | Total agreement |

16 - Antibiotics can cause secondary infections after killing good bacteria present in our organism

| 1 | 2 | 3 | 4 |
| --- | --- | --- | --- |
| Total disagreement |  |  | Total agreement |

17 - Antibiotics can cause allergic reactions.

| 1 | 2 | 3 | 4 |
| --- | --- | --- | --- |
| Total disagreement |  |  | Total agreement |

**Fourth part: awareness about antibiotic resistance**

18–Have you ever heard about antibiotic resistance?

- yes
- no

19 - In particular, have you discussed the problem of antibiotic resistance during degree courses?

- yes
- no

20-Have you ever heard of it outside degree courses? If yes, where have you heard it from? (more than one answer is possible)

- I have never heard about it outside degree course
- General Practitioner
- Television
- Newspaper
- Web
- Other _____________________

21 - Antibiotic resistance is a phenomenon for which a bacterium loses its sensitivity to an antibiotic.

| 1 | 2 | 3 | 4 |
| --- | --- | --- | --- |
| Total disagreement |  |  | Total agreement |

22 - Misuse of antibiotics can lead to a loss of sensitivity of an antibiotic to a specific pathogen.

| 1 | 2 | 3 | 4 |
| --- | --- | --- | --- |
| Total disagreement |  |  | Total agreement |

23 - If symptoms improve before it is completed the full course of antibiotic, you can stop taking it.

| 1 | 2 | 3 | 4 |
| --- | --- | --- | --- |
| Total disagreement |  |  | Total agreement |

**Firth part: attitudes regarding consumption of antibiotics**

24 - Do you usually take antibiotic for cold or sore throat?

- yes
- no

25–Do you usually take antibiotic for fever?

- yes
- no

26 - Do you usually stop taking antibiotic when you start feeling better?

- yes
- no

27 - Do you take antibiotic only when prescribed by the doctor?

- yes
- no

28–Do you keep leftovers antibiotics at home because they might be useful in the future?

- yes
- no

29 - Do you use leftovers antibiotics when you have cold, sore throat or flu without consulting your doctor?

- yes
- no

30–Do you buy antibiotics without a medical receipt?

- yes
- no

31–Have you ever started an antibiotic therapy after a simple doctor call, without a proper medical examination?

- yes
- no
