# Supplementary material for: Antibiotic Use: A Cross-Sectional Survey Assessing the Knowledge, Attitudes and Practices amongst Students of a School of Medicine in Italy
Source: PLoS One. 2015 Apr 1;10(4):e0122476. doi: 10.1371/journal.pone.0122476 (PMC4382153; doi:10.1371/journal.pone.0122476)
Supplement: S1 Table — (DOC) [file pone.0122476.s002.doc]

**Table S1. Knowledge about antibiotics.**

| **Topic** | **Statement** | **Answer** | N (%) |
| --- | --- | --- | --- |
| **Identification of antibiotics** | **Penicillin is an antibiotic** | Strongly agree | 833 (79.6) |
|  |  | Agree | 171 (16.3) |
|  |  | Disagree | 33 (3.1) |
|  |  | Strongly disagree | 10 (1) |
|  | **Aspirin is an antibiotic** | Strongly agree | 5 (0.5) |
|  |  | Agree | 6 (0.6) |
|  |  | Disagree | 32 (3) |
|  |  | Strongly disagree | 1005 (95.9) |
|  | **Paracetamol is an antibiotic** | Strongly agree | 17 (1.6) |
|  |  | Agree | 15 (1.4) |
|  |  | Disagree | 84 (8) |
|  |  | Strongly disagree | 932 (88.9) |
| **Knowledge about antibiotic role** | **Antibiotics are useful for bacterial infections** | Strongly agree | 869 (83) |
|  |  | Agree | 128 (12.2) |
|  |  | Disagree | 31 (3) |
|  |  | Strongly disagree | 19 (1.8) |
|  | **Antibiotics are useful for viral infections** | Strongly agree | 86 (8.2) |
|  |  | Agree | 89 (8.5) |
|  |  | Disagree | 138 (13.2) |
|  |  | Strongly disagree | 734 (70.1) |
|  | **Antibiotics are the same as anti-inflammatory agents** | Strongly agree | 9 (0.86) |
|  |  | Agree | 26 (2.5) |
|  |  | Disagree | 171 (16.3) |
|  |  | Strongly disagree | 841 (80.3) |
| **Knowledge about side-effects** | **Antibiotics can kill “good bacteria” present in our organism** | Strongly agree | 728 (69.5) |
|  |  | Agree | 224 (21.4) |
|  |  | Disagree | 73 (6.9) |
|  |  | Strongly disagree | 23 (2.2) |
|  | **Antibiotics can cause secondary infections after killing good bacteria present in our organism** | Strongly agree | 404 (39.1) |
|  |  | Agree | 417 (40.4) |
|  |  | Disagree | 179 (17.3) |
|  |  | Strongly disagree | 33 (3.2) |
|  | **Antibiotics can cause allergic reactions** | Strongly agree | 754 (72.3) |
|  |  | Agree | 219 (21) |
|  |  | Disagree | 58 (5.6) |
|  |  | Strongly disagree | 12 (1.15) |
| **Knowledge about antibiotic resistance** | **Antibiotic resistance is a phenomenon for which a bacterium loses its sensitivity to an antibiotic** | Strongly agree | 725 (69.8) |
|  |  | Agree | 251 (24.2) |
|  |  | Disagree | 40 (3.8) |
|  |  | Strongly disagree | 23 (2.2) |
|  | **Misuse of antibiotics can lead to antibiotic resistance** | Strongly agree | 903 (86.2) |
|  |  | Agree | 124 (11.8) |
|  |  | Disagree | 14 (1.3) |
|  |  | Strongly disagree | 7 (0.7) |
|  | **It is okay to stop taking antibiotic when symptoms are improving** | Strongly agree | 826 (78.8) |
|  |  | Agree | 166 (15.8) |
|  |  | Disagree | 32 (3.1) |
|  |  | Strongly disagree | 24 (2.3) |
