# Supplementary material for: Antibiotic Use: A Cross-Sectional Survey Assessing the Knowledge, Attitudes and Practices amongst Students of a School of Medicine in Italy
Source: PLoS One. 2015 Apr 1;10(4):e0122476. doi: 10.1371/journal.pone.0122476 (PMC4382153; doi:10.1371/journal.pone.0122476)
Supplement: S2 Table — (DOCX) [file pone.0122476.s003.docx]

**Table S2. Multivariate results on attitudes and behaviors about antibiotic consumption (including the variable “Degree course”) (N = 1,050)**

|  |  | **Do you have leftover antibiotics at home?** | | **Do you usually take antibiotics after a simple doctor call without a proper medical examination?** | |
| --- | --- | --- | --- | --- | --- |
|  |  | **OR (95% C.I.)** | **p** | **OR (95% C.I.)** | **p** |
| **Gender** | **Male** | 1 |  | 1 |  |
|  | **Female** | 0.94 (0.72-1.23) | 0.65 | 1.12 (0.85-1.46) | 0.42 |
| **Age*** | **>18** | 0.92 (0.88-0.97) | < 0.01 | 1.00 (0.95-1.05) | 1.00 |
| **Country of birth** | **Italy** | 1 |  | 1 |  |
|  | **Foreign** | 0.85 (0.42-1.74) | 0.66 | 0.57 (0.25-1.32) | 0.19 |
| **Relatives working in health-related field** | **No** | 1 |  | 1 |  |
|  | **Yes** | 1.81 (1.35-2.43) | < 0.01 | 0.66 (0.50-0.89) | < 0.01 |
| **Degree course** | **Medicine** | 1 |  | 1 |  |
|  | **Health professions** | 0.64 (0.47-0.87) | < 0.01 | 0.60 (0.43-0.83) | < 0.01 |
|  | **Dentistry** | 0.73(0.36-1.47) | 0.38 | 1.18 (0.59-2.38) | 0.64 |
| **Use of antibiotic in the last year** | **No** | 1 |  | 1 |  |
|  | **Yes** | 2.42 (1.85-3.17) | < 0.01 | 1.87 (1.44-2.43) | < 0.01 |
| **Have you ever heard about antibiotic resistance** | **No** | 1 |  | 1 |  |
|  | **Yes** | 0.68 (0.37-1.25) | 0.21 | 0.53 (0.30-0.92) | 0.02 |

* “Age” and “Time spent at university” are considered as continuous variables.
